# Testing the Bed-Blocking Hypothesis: Does Nursing and Care Home Supply Reduce Delayed Hospital Discharges?

**DOI:** 10.1002/hec.3150

**Published:** 2015-03-11

**Authors:** James Gaughan, Hugh Gravelle, Luigi Siciliani

**Affiliations:** Centre for Health Economics, University of YorkYork, UK

**Keywords:** delayed discharges, long-term care, nursing and care homes, bed blocking, substitution

## Abstract

Hospital bed-blocking occurs when hospital patients are ready to be discharged to a nursing home, but no place is available, so that hospital care acts as a more costly substitute for long-term care. We investigate the extent to which greater supply of nursing home beds or lower prices can reduce hospital bed-blocking using a new Local Authority (LA) level administrative data from England on hospital delayed discharges in 2009–2013. The results suggest that delayed discharges respond to the availability of care home beds, but the effect is modest: an increase in care home beds by 10% (250 additional beds per LA) would reduce social care delayed discharges by about 6–9%. We also find strong evidence of spillover effects across LAs: more care home beds or fewer patients aged over 65 years in nearby LAs are associated with fewer delayed discharges. © 2015 The Authors. *Health Economics* Published by John Wiley & Sons Ltd.

## 1. Introduction

Hospital bed-blocking occurs when a patient is medically ready to be discharged and cared for in another setting. Because hospital care is more expensive than nursing or residential home care, bed-blocking is a signal of allocative inefficiency. There is concern about bed-blocking in countries including Australia, Austria, the Netherlands, Sweden and the UK (Brown *et al*., 2011; Mur-Veeman and Govers, 2011; National Audit Office, 2000).

We investigate the extent to which greater supply of nursing and care home beds reduces delays in hospital discharges (i.e. the degree of substitution between formal long-term care (LTC) and health care). Whether policymakers should encourage such increases in supply to reduce delayed discharges depends, inter alia, on the elasticity of the number of delayed discharges with respect to the availability of care home beds. If the elasticity is high, then increase in care home supply will have a significant positive externality on the hospital sector.

The rate at which hospital patients are discharged into a nursing home may depend not only on the supply of beds but also on their price. Unlike health care, which is free or heavily subsidised in most Organisation for Economic Cooperation and Development countries, there is limited insurance for nursing home costs (Cremer *et al*., 2012). Hence, higher prices may prolong search and make patients more reluctant to be transferred to a nursing home and hence increase bed-blocking. If so, policy interventions, which reduce prices for nursing homes (such as encouraging competition, Forder and Allan, 2014) may also have beneficial effects in the hospital sector.

We also explore whether the supply of care homes in a Local Authority (LA) affects delayed discharges in other LAs. This is important for policy. If spillover effects across LAs are negligible, then policymakers will have to pay more attention to variations in care homes availability across LAs, because they will also imply variations in delayed discharges. But variation in provision across LAs may be of less concern if patients are willing to accept a bed in other LAs. Spillover effects across LAs may also raise coordination issues by weakening incentives to expand care home capacity.

To answer our research questions, we first provide a theoretical framework for understanding hospital delayed discharges. The empirical analysis then uses a new English 2009–2013 panel dataset on delayed discharges (Department of Health, 2011a) and a mix of econometric approaches. To control for unobserved heterogeneity at LA level, we use panel-data models, which reduce the risk of omitted variable bias due to time-invariant unobservables correlated with both hospital delays and availability of care homes. Unobserved heterogeneity is likely to be important because LAs differ in needs, geography, population size, policies and controlling political party. We also allow for possible simultaneity bias, arising because social care beds supply, prices and delays in hospital discharges are jointly determined, by instrumenting current social care beds and prices with their 1 or 2-year lagged values. To test for spillover effects across LAs (our second research question), we use spatial econometrics methods, which allow for spatial dependence across geographical units (Moscone and Tosetti, 2014).

We find that delayed discharges do respond to the availability of care home beds. The response is modest: an increase in care home beds of 10% (250 additional beds per LA) would reduce social care delayed discharges by 6–9%. Although their estimated effects are less robustly estimated, higher prices also contribute to increasing delayed discharges.

We also find spillover effects across LAs. Higher availability of care home beds in other LAs reduces delayed discharges, although higher prices in other LAs have no statistically significant effect. Higher population in other LAs increases delayed discharges, suggesting that patients are willing to cross LA boundaries to secure a care home bed.

### 1.1. Related literature

There is an extensive literature on substitution between informal care and formal long-term care, but few studies on substitution between care homes (formal long-term care) and delayed hospital discharges (health care).^1^ Forder (2009) used cross-section electoral ward level data in England and found that increasing spending on care homes by £1 reduces hospital expenditure by £0.35. Holmås *et al*. (2010) investigated the effect of fining owners of long-term care institutions who prolong length of stay at hospitals in Norway. Øien *et al*., (2012) investigated the effect of long-term financing on the composition of long-term services at municipality level in Norway. Picone *et al*. (2003) investigated the simultaneous determinants of hospital length of stay and discharge destination of US Medicare patients following hip fracture, stroke or heart attack. The study that is closest to ours is Fernandez and Forder (2008) who use 1998/1999 and 1999/2000 data for English LAs and find that LAs with more home help hours, and nursing and residential care beds, had a lower rate of hospital delayed discharges and lower emergency readmission rates.

Our study makes several innovations. Our theoretical model augments stochastic queuing theory with endogenous demand (baulking) to explain social care market equilibria with positive waiting times for care home places. Our data set is recent (2009–2013) and has measures for hospital delays, which distinguish between delayed patients and the number of days of delay, and between total delays and those due to social care. We also have data on the numbers of beds, prices and quality for all nursing and care homes. We exploit the panel-data to control for endogeneity due to unobserved heterogeneity at LA level and to construct instruments for the potentially endogenous supply of social care beds and prices. We also use spatial econometrics regressions to test for spillover effects across LAs.

## 2. Institutional Setting

Hospitals and nursing and care homes in England have different organisational arrangements and funding. Hospital care is provided by 164 public hospitals paid by a mix of nationally set prospective prices and block contracts negotiated with local health authorities. National Health Service patients do not pay for hospital care. By contrast, there are over 18 000 providers of social care (nursing and residential homes) (Laing and Buisson, 2014), which are a mix of for-profit, nonprofit and public organisations. Around 60% of users pay for social care (Forder, 2007), with those on low income or wealth being subsidised. LAs provide means tested personal social services, including home help.

Long-standing concerns about the coordination of health and long-term care for patients discharged from hospital led to the Community Care (Delayed Discharges) Act (2003).^2^ The Act requires LAs and hospitals to collaborate around the discharge of patients from hospital. LAs must reimburse hospitals for delayed discharges for which they are solely responsible.

## 3. A Model of Patients Waiting for Hospital Discharge

We observe the number of patients waiting for hospital discharge at a census date. Assume that all patients with a delayed hospital discharge (i.e. medically ready for discharge but still in hospital) are waiting to find a place in a nursing home. We require a model that explains why patients are waiting given that nursing homes could raise prices and reduce waiting times. To explain such equilibria with positive waiting times, we assume that demand and patient length of stay in a nursing home are uncertain: we use a stochastic queuing model with endogenous demand (baulking).

Suppose, initially, that there is a single nursing home with *k* beds and that the number of patients who complete their hospital treatment and are ready to be discharged follows a Poisson distribution with mean rate γ.^3^ A proportion *θ* of these patients wish to enter a nursing home, so that the flow rate of demand for a nursing home place is also Poisson distributed with mean *λ* = γ *θ* (the arrival rate). Patient length of stay in the nursing home is exponentially distributed with a mean of 1/*kμ*, where *μ* is the ‘service’ rate in bed.^4^ The expected waiting time (delay) before a nursing-home bed becomes available depends on the number waiting in hospital and the rate at which beds become available. The expected waiting time is^5^


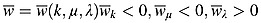
(1)

By Little's Law (Little, 1961), the expected number of patients waiting for a place is


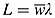
(2)

We assume that patients know the expected waiting time and that patient expected utility from a nursing home place after a delay of 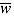 is 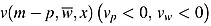 where *m* is income, *p* is the price of a care home bed and *x* is a vector of patient characteristics. Utility from the alternative of discharge to the patient's home is *v^o^*(*m*,*x*). The proportion of patients *θ* who opt for a nursing home place (i.e. who have 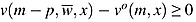) depends on the expected delay 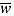, nursing home price *p*, and the joint distribution of income and other characteristics:


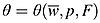
(3)

where *F* is a vector of parameters affecting patient preferences and characterising the joint distribution of *m* and *x*. Ceteris paribus, higher prices and longer expected waiting times reduce the proportion of patients who demand a nursing home bed: *θ*_*p*_ < 0 and 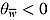.

The arrival rate for patients demanding a nursing home bed satisfies the implicit function


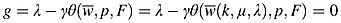
(4)

which can be solved explicitly for the arrival rate as


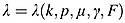
(5)

with


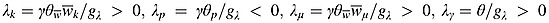
(6)

where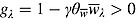.

The care home chooses beds and price to maximise expected profit


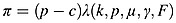
(7)

so that equilibrium beds supply *k*(*μ*, *γ*, *F*) and price *p*(*μ*, *γ*, *F*) are also functions of the exogenous factors entering patient preferences and the cost function.^6^ From Little's Law, the expected number of patients waiting for discharge to a nursing home is


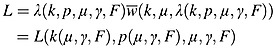
(8)

We want to estimate the effects of beds and prices on delay and so estimate 8, rather than the reduced form *L^o^*(*μ*,*γ*,*F*). But, as 8 makes clear, in the empirical analysis, we need to take account of the fact that prices and beds are endogenous, so that prices, beds and the number waiting are jointly determined. Section 4 discusses how we allow for this using LA effects and instruments for prices and beds.

### 3.1. Comparative statics

The expected number waiting *L* is decreasing in nursing home prices: a *ceteris paribus* increase in *p* reduces the proportion of patients who opt for nursing homes (*λ_p_* < 0) and the expected wait


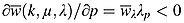
(9)

Thus, both parts of 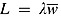 are reduced by an increase in *p* and ∂*L*/*dp* < 0.

The effect of an increase in the number of beds *k* is ambiguous:


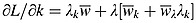
(10)

because it increases demand via its effect on waiting time, but it also reduces the waiting time so that 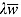 could increase or decrease. It is possible to show that the number waiting increases or falls depending on whether the demand for nursing home places is elastic or inelastic with respect to expected waiting time. If demand is inelastic, the expected number waiting will fall.^7^

Conditional on *p* and *k*, an increase in throughput (*μ*) in nursing homes also has an ambiguous effect because it affects waiting time directly and via its effect on demand. LAs with a larger or sicker population will have higher *γ* and will have more patients waiting for discharge because 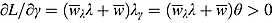.

### 3.2. Extensions to the model

We can generalise the model for there to be more than one nursing home. Let the proportion of patients choosing nursing home *h* be 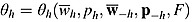, where 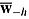 and **p**_− *h*_ are vectors of expected waiting times and prices at other nursing homes. The expected number waiting for a place at home *h* is again determined by Little's Law as 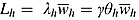 where 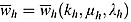 and so the expected total number waiting to be discharged to a nursing home is


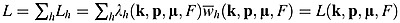
(11)

where **k**, **p**, **μ** are vectors of beds and so on in all homes.^8^

Suppose that patients (or their relatives) search among the set of nursing homes to find an expected utility maximising (net of search costs) combination of price and expected waiting time. Having chosen a home, the patient then waits in hospital for a place. The search may start before the patient is medically ready for discharge, but if it carries on after this date, then the total delay will be greater the longer the patient searches. We do not model the length of search explicitly but, plausibly, we expect that patients will search for longer the greater the dispersion in the prices and waiting times.

## 4. Econometric Models

We use LA-level data and estimate three types of model based on 11.

### 4.1. Panel data

Our first regression model is


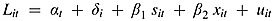
(12)

where *L_it_* is a measure of hospital delayed discharges for patients resident in LA *i* in year *t*, *s_it_* is a vector measuring supply of care homes in LA *i* in year *t* (total beds and average bed prices) and *x*_*it* is_ a vector of control variables, such as the elderly population. *δ_i_* is a LA effect, which controls for unobserved heterogeneity at LA level and *α_t_* is the year effect. We estimate 12 by random effects (RE) with robust standard errors and clustering on LAs. To test whether the random-effects model is preferred to a fixed-effects model, we add the mean of the time-varying variables to 12 and test for its significance (Mundlak, 1978; Wooldridge, 2010). If the means are jointly insignificant, then the random-effects specification is preferred.

### 4.2. Spatial effects

To test whether the effect of care homes supply spills over across LA boundaries, we estimate spatial econometric models


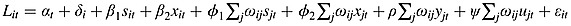
(13)

where *L_it_*, *s_it_* and *x_it_* are specified as in 12. *ω_ij_* ≥ 0 is a distance (spatial) weight. The coefficients *φ* on the spatially lagged regressors tests whether supply of care homes or covariates such as elderly population in nearby LAs affect delayed discharges in a given LA, that is, they test for spillovers. The coefficient *ρ* on the spatially lagged dependent variable allows for higher delays in nearby LAs to be associated with more delayed discharge in a given LA. For example, unobserved local demand factors could affect delays in the LA and in its neighbouring LAs. The inclusion of the spatially lagged dependent variable therefore helps to control for omitted-variable bias. The coefficient *ψ* on the spatial error term allows for correlation between delay in the LA and the error terms in neighbouring LAs.

We use four versions of 13. The simplest is the spatially lagged Xs (SLX) model, which applies spatial weights to only the explanatory variables. The spatially autoregressive model includes the spatially lagged dependent variable. The spatial Durbin model has spatial lags of the dependent and independent variables. The spatial Durbin error model includes spatially lagged errors and independent variables.^9^ We row-standardise the weight matrix.^10^

### 4.3. Instrumental variables

As we noted in discussing the theoretical model, beds supply, prices and delayed discharges are jointly determined in equilibrium. Thus, failing to include variables that affect demand will bias the estimated effects of beds and prices on delay. If these omitted variables are time invariant, then the inclusion of LA effects in the model will remove potential bias. But the omitted variables may be time varying or delays may affect supply (simultaneity) so that potential bias is not removed by the LA effects in the estimated models. We therefore also estimate models in which we instrument beds and prices with their 1 or 2-year lagged values.

## 5. Data

### 5.1. Dependent variables

The delays data are from the ‘Acute and Non-Acute Delayed Transfers of Care’ dataset (Department of Health, 2011a) for 5 years 2009–2013. The Community Care (Delayed Discharges) Act (2003) requires LAs to reimburse National Health Service hospitals for each day an acute patient's discharge is delayed if the sole reason for that delay is the responsibility of the LA Social Services department either in making an assessment of the patient for community care services or in providing those services. Hospitals have to keep records of the number of patients delayed, days of delay and the institution responsible for the delays.^11^

The dataset records delays in the transfer of patients from hospital care to social care in England by each of 147 LAs. The relevant LA is the council with responsibility for adult social care where the patient resides. We use two dependent variables (i) *delayed patients*: the number of patients who are ready to be discharged from hospital into social care but have not been discharged at midnight on the last Thursday of each month, averaged over the year; and (ii) *days delayed* during the month experienced by all patients with delayed discharges, not just those waiting at census date, averaged over the year.

The data distinguish between delays attributed to the hospital, to social care (which is the responsibility of the LA in which the patient lives) and to both. To allow for the possibility of misclassification, we estimated models for (i) delays officially attributed to social care and (ii) all delays.^12^ The results were qualitatively very similar and so in the text, we report results for delays officially attributed to social care, relegating results for models of all delays to the online Appendix in Supporting Information.

### 5.2. Supply of long-term care

We measure for each LA the number of care home beds and the average price charged by care homes and their quality rating. Data on individual care (residential/nursing) homes were aggregated to LA level by mapping the postcode of each provider to a LA. We include only providers whose ‘primary client’ is people aged 65 years and over. We use Laing and Buisson data (Laing and Buisson, 2014) to obtain care homes prices per week and take the unweighted average price of beds across eight categories.

As a robustness test, we include the quality of care homes as a covariate in some models. The Care Quality Commission rates care homes as Poor, Adequate, Good or Excellent. We measure the quality of care homes in an LA as the percentage rated Excellent. The data are only available for 1 year (2010), and we use this value for all years.

### 5.3. Control variables

We control for the population within each LA who are aged 65 years and over using Office of National Statistics (Office of National Statistics, 2011) mid-year population estimates for 2009–2013. We also use the percentage of people aged 65 years and over receiving social security benefits as a control for deprivation in a LA. This variable is measured only for 2010 and treated as time-invariant. To control for population health, we use the number of deaths among people aged 65 years and over. We use 2-year lagged values of these variables to avoid the ‘bad control’ problem (Angrist and Pischke, 2009).

## 6. Results

### 6.1. Descriptive statistics

Table I shows that 28 patients are delayed at the monthly census day in an average LA of which 8.5 are attributed solely to social care. In an average calendar month, 785 bed days are lost because of patients not being discharged when ready, of which 236 days are classified as the responsibility of social care. The average LA has population aged 65 years and over of 60 000 and 2500 residential or nursing home beds. The average LA price for a week of care is £550, although it can reach over £1000.

**Table I:** Summary statistics

Variable	Mean	SD (Overall)	SD (Between)	SD (Within)	Min	Max
Delayed patients (all patients)	28.44	28.12	27.17	7.522	1.08	155.67
Days of delay (all patients)	784.9	816.4	788.8	218.6	36.33	4911
Delayed patients attributed to social care	8.505	11.39	10.83	3.627	0	100.8
Days of delay attributed to social care	236.1	330.9	316	101.1	0	2908
Care homes beds	2506	2335	2327	263.4	233	12 496
Care homes price	546	112.7	107.3	35.37	364.8	1081
Population over 65 years old	59 700	52 460	52 500	3295	7455	286 310
% care homes rated excellent in 2010	20.34	12.38	12.42	0	0	70
% age 65+ years on income benefit	20.89	7.86	7.88	0	7.26	51.98
Deaths in population over 65 years	2603	2195	2200	77.02	281	11 503

SD, standard deviation.

Data are for 147 Local Authorities over 2009–2013. Mean, min and max are over 5 years. Delayed patients: number waiting for discharge on monthly census date. Days of delay: total days of delay experienced by all delayed patients during a month. Delayed patients and delayed days are averages of monthly data over the year. Deaths are lagged by 2 years.

Preliminary investigation suggested that we could use a specification in which both the dependent and explanatory variables are measured in logs so that reported coefficients are elasticities.^13^,^14^

### 6.2. Regression results

Table II reports results from the baseline RE and SLX models of patients whose delays are attributed officially to social care. We estimate models for the number of patients delayed on a census day (patients delayed) in an average month and the total number of bed days lost because of delays (days of delay). All models in Table II pass the Mundlak and Hausman tests, supporting the use of random rather than fixed-effects specification. The SLX models include spatial lags of beds and of the elderly population in other LAs.^15^ Results for models where the dependent variables are patients delayed or days delayed for all causes are very similar to those where delays are officially attributed to social care. They are reported in the online Appendix in Supporting Information (Table A1).

**Table II:** Delayed discharges attributed to social care

	Patients delayed				Days of delay			
	RE		SLX		RE		SLX	
	coefficient	*p*	coefficient	*p*	coefficient	*p*	coefficient	*p*
Care homes beds	−0.670***	(0.001)	−0.578***	(0.008)	−0.921***	(0.001)	−0.784***	(0.007)
Care homes price	0.697**	(0.031)	0.603	(0.156)	0.983**	(0.014)	0.851*	(0.098)
Population of 65+ years	1.738***	(0.000)	1.599***	(0.000)	2.229***	(0.000)	2.020***	(0.000)
2010	−0.0851**	(0.031)	−0.161***	(0.001)	−0.208***	(0.001)	−0.323***	(0.000)
2011	−0.174***	(0.006)	−0.193***	(0.007)	−0.185**	(0.027)	−0.214**	(0.022)
2012	−0.313***	(0.000)	−0.254**	(0.012)	−0.320***	(0.001)	−0.234*	(0.052)
2013	−0.423***	(0.000)	−0.480***	(0.000)	−0.404***	(0.000)	−0.493***	(0.001)
Beds spatial lag			−2.844***	(0.003)			−4.262***	(0.005)
Pop 65+ years spatial lag			4.878***	(0.001)			7.332***	(0.002)
Constant	−15.54***	(0.000)	−45.08***	(0.000)	−17.56***	(0.000)	−62.10***	(0.000)
*R*^2^	0.488		0.524		0.441		0.485	
Mundlak Test	3.852	0.278	4.353	0.500	2.649	0.449	3.045	0.693
Hausman Test	5.671	0.579	5.773	0.762	4.983	0.662	4.359	0.886

Dependent variables are for delays officially attributed to social care. Dependent and continuous explanatories are in logs. All models are estimated with random effects and cluster robust standard errors. Spatial models: SLX (Spatially lagged Xs). Observations: 735 = 5 × 147.

* *p* < 0.1;

** *p* < 0.05;

*** *p* < 0.01.

The coefficient on beds in the LA is significant and negative in all the random-effects models. Allowing for spatial lags reduces significance and the magnitude of the coefficient somewhat but the beds coefficient is always highly significant. The coefficient on prices is much more variable across the models. It has the expected positive effect but is only significant at 5% when spatial lags are not included.

The spatially lagged beds and population are statistically significant with the expected negative sign on beds and positive sign on the elderly population in other LAs. The coefficient on spatially lagged beds is much larger than the coefficient on beds in the LA. This is to be expected because there will be a much larger supply of beds in the LAs surrounding an LA than in it.

### 6.3. Other spatial specifications

We also investigated variants of the spatial specification 13. The spatial Durbin error model includes spatially lagged errors as well as spatially lagged beds and elderly population. The spatial Durbin model adds spatially lagged dependent variable to the spatial lags of beds and elderly population, and the spatially autoregressive model has spatial lags of the dependent variable rather than spatial lags of beds and elderly population. The estimated coefficients are similar to those from the models with spatially lagged beds and elderly population in Table II (Appendix Tables A3 and A4 of Supporting Information).

### 6.4. IV models

The first two models of Table III provide the results from instrumental variable (IV) models in which beds and prices are instrumented with their 1-year lagged values.^16^ The *F* statistics on the instruments from the first-stage regressions are very large, indicating that lagged beds and prices are strong instruments. The coefficient on beds is generally larger (in absolute value) compared with Table II.

**Table III:** Patients delayed and days of delay (attributed to social care)

	IV models				Augmented models			
	Patients delayed		Days of delay		Patients delayed		Days of delay	
Care homes beds	−0.807**	(0.015)	−0.913**	(0.040)	−0.689***	(0.003)	−1.010***	(0.001)
Care homes price	1.165**	(0.025)	1.452**	(0.039)	1.226***	(0.009)	1.413***	(0.007)
Population of 65+ years	1.818***	(0.000)	2.134***	(0.000)	0.765	(0.107)	0.285	(0.674)
2010	−0.187***	(0.002)	−0.352***	(0.000)	−0.191***	(0.000)	−0.353***	(0.000)
2011	−0.249***	(0.001)	−0.273***	(0.007)	−0.182**	(0.024)	−0.131	(0.253)
2012	−0.348***	(0.001)	−0.330**	(0.025)	−0.230*	(0.056)	−0.0821	(0.602)
2013	−0.598***	(0.000)	−0.617***	(0.000)	−0.460***	(0.001)	−0.307	(0.110)
SD care homes price					−0.00553	(0.952)	0.0920	(0.549)
% care homes rated excellent					0.000993	(0.824)	−0.00135	(0.840)
% 65+ years on income benefit					0.445***	(0.006)	0.580***	(0.002)
Price*(% 65+ years on income benefit)					−0.0659***	(0.009)	−0.0877***	(0.002)
Deaths in population 65+ years					1.088**	(0.035)	2.116***	(0.008)
Beds spatial lag	−2.385**	(0.013)	−3.950***	(0.005)	−2.931***	(0.001)	−4.514***	(0.002)
Population 65+ years spatial lag	4.828***	(0.000)	7.464***	(0.001)	5.317***	(0.000)	7.815***	(0.000)
Constant	−52.02***	(0.000)	−69.76***	(0.000)	−60.83***	(0.000)	−77.30***	(0.000)
*R*^2^	0.523		0.486		0.585		0.541	
*F* test (beds)	102.37	(0.000)	98.51	(0.000)				
*F* test (price)	128.61	(0.000)	123.37	(0.000)				
*F* test (beds spatial lag)	1364.27	(0.000)	1308.15	(0.000)				
Hausman Test	2.388	0.984	1.810	0.994				
Mundlak Test					17.88	0.0221	13.82	0.0865

SD, standard deviation.

IV and augmented models.

Dependent variables are for delays officially attributed to social care. Dependent variable and continuous explanatories are in logs. All models are estimated with random effects and cluster robust standard errors. *F* tests are for the joint significance of the instruments in each first-stage model. The instruments are 1 year lag of care homes beds, 1 year lag of care homes price and 1 year spatially lagged care homes beds. Percentage of 65+ years on income benefit is the proportion of the population aged 65 years and over who are receiving income support in 2010. Observations: 735 = 5 × 147.

* *p* < 0.1;

** *p* < 0.05;

*** *p* < 0.01.

### 6.5. Sensitivity analyses

We estimated models with additional explanatory variables suggested by the theoretical model and the institutional structure. The quality of nursing and care homes in the LA may affect demand for care homes. We therefore included the percentage of care homes in the LA, which were rated Excellent in 2010 as a quality measure.

We also added the LA mortality rate for the over 65 population. To allow for the possibility that longer delays in hospital could affect mortality, we use the 2-year lag of death rate of population over 65 years. Mortality has an *a priori* ambiguous effect. LAs with sicker populations may have more demand for social care beds (corresponding to higher γ in the theory model), and this will increase the number waiting. But, with higher death rates, length of stay (1/*μ* in the theory model) in care homes may be shorter so that more beds become available in a given period, and the theory model shows that this could reduce or increase the number waiting.

Patients with low income or wealth are entitled to subsidies to reduce the cost of a care home bed. We therefore included the proportion of the population aged over 65 years who were in receipt of income-related social security benefits in 2010. We incorporate this variable in the model in two ways. First, because poorer individuals are more likely to be in poor health, we could regard it as another proxy for morbidity, and we therefore add it to the model. Second, the higher the proportion of poor elderly in the LA, the less sensitive will demand for care homes are to the price of beds. We therefore add the interaction of income deprivation with beds price to the model.

Finally, we conjectured when discussing extensions to the theory model that patients (or their relatives) are likely to spend longer searching for a care home bed the greater the dispersion of prices. We therefore include the standard deviation of care home prices in the LA to the model.

The results for the third and fourth models in Table III show that the inclusion of these additional variables does not qualitatively affect the estimates of the effects of care home beds and prices. The Mundlak tests suggest, however, that the RE specification may be less appropriate for these augmented models than for the baseline models. Quality rating and the standard deviation of care home prices are always insignificant. Higher needs, as proxied by mortality, increase delays (but are significant only when delays are attributed to social care; see Table A2 for all delays). The effects of income deprivation are as anticipated. The main effect of deprivation (as a proxy for morbidity) is to increase delays, and the effect of price on delay is reduced in absolute value when patients are more income deprived.

### 6.6. Quantitative effect

The theory model indicated that the effect of an increase in supply on delays is in principle indeterminate. Higher supply reduces the expected waiting time but also increases demand. Whether the number waiting increases or falls depends on whether the demand for nursing home places is elastic or inelastic with respect to expected waiting time. Our results generally suggest that delays reduce with larger supply implicitly suggesting that the demand for nursing home places is relatively inelastic.

Our preferred models in Table II yield an elasticity of the number of patients delayed with respect to beds supply of −0.58 to −0.67. Thus, an increase in care home beds of 10% (from 2500 to 2750 in an average LA) would reduce the number of patients delayed each month by 5.8-6.7%. Given a monthly average number of 8.5 delayed patients, this corresponds to a reduction of less than one patient per month (0.49–0.57 patients) in an average LA. In terms of delays measured in hospital bed days in Table II, a 10% increase in home-care beds would reduce delayed bed days by 7.8–9.2%, which, given an average of 236 delayed bed days in a month, corresponds to a reduction of 18–22 days per month in an average LA. The quantitative effect appears therefore to be relatively modest. When *all* delays are used as dependent variable, the elasticities with respect to beds are smaller −0.36 to −0.44 for delayed patients and −0.32 to −0.39 for delayed bed days (Appendix Table A1 in Supporting Information). Given a monthly average number of 28.44 (785) delayed patients (bed days), this corresponds to a reduction of 1–1.2 patients (25–30 bed days) per month in an average LA. The quantitative effect appears therefore to be larger when the more inclusive definition of delays is used but remains qualitatively similar and still relatively modest.^17^ The small effect on delays suggests that increasing the supply of care home beds will not be cost reducing,^18^ although a full evaluation of such policies would need to take account of the possible gains to patients from a more rapid transfer to a more appropriate care setting and the use of the extra social care beds by people who enter care-homes directly rather than via hospital.

## 7. Conclusions

Coordination between the health and long-term care sectors is critical to address concerns about hospital bed-blocking. This study has investigated the extent to which expanding the supply of nursing and care home beds can reduce delayed discharges. The results suggest that delayed discharges in hospitals do respond to the availability of care home beds but that the response is relatively modest: an increase in care home beds of 10% (250 additional beds per LA) would reduce social care delayed discharges by 6–9%. Although less robustly estimated, we find some evidence of positive effect of care home prices on delayed discharges. These may arise because patients spend longer searching in markets with higher average prices. Policies aimed at encouraging competition across care homes and at reducing prices may therefore bring further reductions of hospital delays.

We find spillover effects across LAs with respect to both care home beds and elderly population. Higher availability of care homes in other LAs reduces delayed discharges. Similarly, higher population in other LAs increases delayed discharges. This suggests that patients are willing to cross boundaries in order to secure a bed in a care home.

A key implication is that policies aimed at specific LAs need to take account of these spillovers, which could otherwise lead to free riding and ‘races to the bottom’ in the absence of coordination across authorities. For example, a LA would have a weaker incentive to encourage an expansion of care home capacity if some of the benefits in terms of reductions in delayed discharge accrue to neighbouring LAs or if the needs of the elderly population of a LA can be satisfied by neighbouring capacity. The presence of such spillover effects, with patients in one LA willing to accept beds in nearby LAs, implies that inequalities in care home availability across LAs may be of less concern than the total supply of care home beds.

## Notes

See Norton (2000), Grabowski *et al*. (2012), Pestieau and Ponthière (2012), Cremer *et al*. (2012) and Siciliani (2013) for comprehensive literature reviews on the economics of long-term care.

Baumann *et al*. (2007), Department of Health (2003, 2011b), House of Commons, Committee of Public Accounts (2003), National Audit Office (2000).

This is the number in the Local Authority, which is our unit of analysis. Patients may be in several hospitals serving the Local Authority's patients.

For example, suppose that patients exit a nursing home only on death.

See Gross *et al*. (2008) for the complicated expression for .

If the queueing model was deterministic, as in Lindsay and Feigenbaum (1984), with demand function *D*(*p*,*w*) and output *S*, then waiting time *w*(*p*,*S*) is determined by *D*(*p*,*w*) − *S* = 0 for *w* > 0 and *D*(*p*,0) − *S* ≤ 0 for *w* = 0, with *w_p_* < 0, *w_S_* < 0 if *w* > 0. It can never be profit maximising to have a positive queue: if *w* > 0, the care home can raise price, keeping output constant and letting the waiting time fall, thereby increasing profit because revenue is increased and costs unchanged. Thus, nonstochastic waiting time models cannot explain the existence of positive queues, that is, of people waiting to be discharged.

Intuitively, Little's Law  is analogous to the expression for revenue (price times demand) and revenue falls if price is reduced if and only if demand is inelastic with respect to price. There is no evidence on the effect of waiting times on the demand for nursing home places, but most studies of the effect of hospital waiting times report that demand for hospital care is inelastic with respect to hospital waiting times (Gravelle *et al*., 2003; Martin *et al*., 2007).

The arrival rates *λ_h_* and expected waiting times are found by solving  and  simultaneously for all *h*. The equilibrium beds *k_h_* and prices *p_h_* are found as the Nash equilibrium where each nursing home maximises profit given *λ*_*h*_ = *λ*_*h*_(**k**, **p**, **μ**, *F*) and .

We estimate 13 by maximum likelihood (Moscone and Tosetti, 2014).

Define *d_ij_* as the distance between LA *i* and LA *j*. The weights are given by *ω_ij_* = 0, if *i* = *j*, *ω_ij_* = (*d_ij_*^−1^)/(∑_j_ *d_ij_*^−1^) if *i* ≠ *j*. The inverse distance specification gives a lower weight to the delayed discharges of LAs that are more distant from Local Authority *i*. This row-standardisation permits us to interpret *W L* as a weighted average delayed discharges across LAs, where the weights are inversely related to the distance between LAs centroids. Similarly, we can interpret *Ws* as the weighted average LTC supply and *Wx* as the average population or demand shifter.

A patient is considered delayed if ‘a clinical decision has been made that patient is ready for transfer and a multidisciplinary team decision has been made that patient is ready for transfer and the patient is safe to discharge/transfer’. In this context, a multidisciplinary team includes ‘nursing and other health and social care professionals caring for that patient in an acute setting’. A delay can only be attributed to social care if the relevant Trust (hospital) notifies the relevant Local Authority that a patient may require community care and 24 hours notice of the actual need of that care. Delay may be also attributed to both National Health Service and the Local Authority or to the National Health Service. There is formal procedure for disputes.

Delays attributed to social care can include, in addition to those waiting for a bed in a care home, patients who are to be discharged to their own home but are delayed because their LA Social Services department has not yet made arrangements for a care package to be provided at home or for necessary equipment or adaptions to be supplied. Because some delays are attributed both to the NHS and to the LA, the all delays category will include all delays solely or partly attributable to social care. Thus, while both types of dependent variable will include delays for patients who are waiting for a place in a nursing or care home, they will also include delays for patients who are to be discharged to their home. This measurement error in the dependent variable will add noise to the model but should not lead to bias in the estimated coefficients.

In these initial investigations, the link test rejected specifications with the dependent and explanatories measured on the raw scale and with the dependent measured on the raw scale and the explanatories on the log scale. It did not reject the specification with both dependent and explanatories measured on the log scale. This specification also passed the RESET using a quartic function of the predicted dependent variable. Details are available from the authors.

For variables that have 0 values for some LAs, we use the inverse hyperbolic sine transformation  where z is the raw variable (Burbidge *et al*., 1988). *y* ≈ ln *z* + ln 2 for *z* > 0, so that the coefficients can still be interpreted as elasticities.

We also estimated models, which included the spatial lag of prices. This variable made little difference and was never significant. Results are available from the authors.

See Table A2 for the results when delays for all causes are included. Results from models using 2-year lagged values as instruments were very similar and are in Table A5. Table III includes spatial lags of beds and population. The results are similar if the spatial lags are excluded (Table A6).

Effects are only slightly larger when the IV results are used.

Using the average price of a care home bed (£546 per week) as a measure of the average cost, a 10% increase in beds in the average Local Authority (250 beds) would cost about £546*4*250 = £546 000 per month. Assuming a reduction in hospital bed days of 30 per month and a hospital hotel cost of £150 per day, a 10% increase in care home beds for a month would reduce National Health Service costs by £4500.
