# Supplementary material for: Testing the Bed-Blocking Hypothesis: Does Nursing and Care Home Supply Reduce Delayed Hospital Discharges?
Source: Health Econ. 2015 Mar 11;24(Suppl 1):32–44. doi: 10.1002/hec.3150 (PMC4406135; doi:10.1002/hec.3150)
Supplement: Supplementary file 1 [file hec0024-0032-sd1.docx]

**For Supplementary Information in publisher website**

**Appendix Tables**

**Table A1. Delayed Discharges (all delays)**

|  | Patients Delayed | | | | Days of Delay | | | |
| --- | --- | --- | --- | --- | --- | --- | --- | --- |
|  | RE | | SLX | | RE | | SLX | |
|  | coef | p | coef | p | coef | p | coef | p |
| Care-homes beds | -0.436*** | (0.001) | -0.357** | (0.013) | -0.396*** | (0.006) | -0.321** | (0.044) |
| Care-homes price | 0.149 | (0.504) | 0.0170 | (0.951) | 0.0938 | (0.688) | -0.0101 | (0.972) |
| Pop 65+ | 1.498*** | (0.000) | 1.401*** | (0.000) | 1.496*** | (0.000) | 1.395*** | (0.000) |
| 2010 | -0.0379 | (0.167) | -0.0778** | (0.014) | -0.107*** | (0.000) | -0.156*** | (0.000) |
| 2011 | -0.0990** | (0.031) | -0.0998** | (0.043) | -0.0879* | (0.059) | -0.0953* | (0.054) |
| 2012 | -0.161*** | (0.002) | -0.108 | (0.105) | -0.0933* | (0.071) | -0.0435 | (0.500) |
| 2013 | -0.184*** | (0.002) | -0.194** | (0.010) | -0.103* | (0.082) | -0.129* | (0.089) |
| Beds spatial lag |  |  | -1.724*** | (0.004) |  |  | -1.956*** | (0.003) |
| Pop 65+ spatial lag |  |  | 2.768*** | (0.003) |  |  | 3.254*** | (0.002) |
| Constant | -10.68*** | (0.000) | -26.15*** | (0.000) | -7.364*** | (0.000) | -26.39*** | (0.000) |
| R^2^ | 0.662 |  | 0.675 |  | 0.653 |  | 0.67 |  |
| Mundlak Test | 4.360 | 0.225 | 3.118 | 0.682 | 7.393 | 0.0604 | 7.057 | 0.216 |
| Hausman Test | 5.509 | 0.598 | 3.877 | 0.919 | 8.954 | 0.256 | 7.794 | 0.555 |

Dependent variables are for all delays. Dependent variables and continuous explanatories are in logs. All models are estimated with random effects and cluster robust standard errors. Spatial models: SLX (Spatially Lagged Xs). Observations: 735 = 5x147. *p<0.1, **p<0.05, ***p<0.01.

**Table A2. Patients Delayed and Days of Delay (all delays). IV and Augmented Models**

|  | IV models | | | | Augmented models | | | |
| --- | --- | --- | --- | --- | --- | --- | --- | --- |
|  | Patients delayed | | Days of delay | | Patients delayed | | Days of delay | |
| Care-homes beds | -0.499** | (0.010) | -0.421** | (0.032) | -0.419*** | (0.006) | -0.386** | (0.022) |
| Care-homes price | -0.0224 | (0.947) | 0.0634 | (0.865) | 0.334 | (0.289) | 0.328 | (0.332) |
| Pop 65+ | 1.549*** | (0.000) | 1.496*** | (0.000) | 1.031*** | (0.001) | 1.074*** | (0.003) |
| 2010 | -0.0783* | (0.068) | -0.160*** | (0.000) | -0.0940*** | (0.002) | -0.173*** | (0.000) |
| 2011 | -0.0980* | (0.061) | -0.104* | (0.057) | -0.101* | (0.088) | -0.0999 | (0.104) |
| 2012 | -0.104 | (0.155) | -0.0565 | (0.465) | -0.108 | (0.200) | -0.0481 | (0.579) |
| 2013 | -0.191** | (0.019) | -0.147* | (0.093) | -0.194* | (0.056) | -0.137 | (0.197) |
| SD Care-homes price |  |  |  |  | 0.0360 | (0.533) | 0.0339 | (0.630) |
| % care-homes rated excellent |  |  |  |  | -0.00194 | (0.534) | -0.00275 | (0.448) |
| % 65+ on income benefit |  |  |  |  | 0.192 | (0.113) | 0.302** | (0.015) |
| Price*(% 65+ on income benefit) |  |  |  |  | -0.0276 | (0.146) | -0.0449** | (0.020) |
| Deaths in pop 65+ |  |  |  |  | 0.506 | (0.177) | 0.458 | (0.257) |
| Beds spatial lag | -1.654** | (0.018) | -1.863** | (0.010) | -1.650*** | (0.005) | -1.889*** | (0.003) |
| Pop 65+ spatial lag | 2.748** | (0.010) | 3.258*** | (0.003) | 2.867*** | (0.001) | 3.331*** | (0.000) |
| Constant | -26.74*** | (0.000) | -27.90*** | (0.000) | -33.32*** | (0.000) | -35.78*** | (0.000) |
| R^2^ | 0.675 |  | 0.671 |  | 0.71 |  | 0.705 |  |
| F Test (Beds) | 107.51 | (0.000) | 98.68 | (0.000) |  |  |  |  |
| F Test (Price) | 135.59 | (0.000) | 123.6 | (0.000) |  |  |  |  |
| F Test (Beds spatial lag) | 1438.84 | (0.000) | 1310.54 | (0.000) |  |  |  |  |
| Hausman Test | 2.429 | 0.983 | 4.927 | 0.841 |  |  |  |  |
| Mundlak Test |  |  |  |  | 9.856 | 0.275 | 14.73 | 0.0646 |

Dependent variables are for all delays. Dependent variable and continuous explanatories are in logs. All models are estimated with random effects and cluster robust standard errors. F tests are for the joint significance of the instruments in each first stage model. The instruments are one year lag of care-homes beds, one year lag of care-homes price, and one year spatially lagged care-homes beds. % 65+ on income benefit is the proportion of the population aged 65 and over who are receiving income support in 2010.Observations: 735 = 5x147. *p<0.1, **p<0.05, ***p<0.01.

**Table A3. Delayed Discharges attributed to social care. Alternative Spatial Models**

|  | Patients Delayed | | | | | | Days of Delay | | | | | |
| --- | --- | --- | --- | --- | --- | --- | --- | --- | --- | --- | --- | --- |
|  | SDEM | | SAR | | SDM | | SDEM | | SAR | | SDM | |
|  | coef | p | coef | p | coef | p | coef | p | coef | p | coef | p |
| Care-homes beds | -0.581*** | (0.007) | -0.645*** | (0.001) | -0.567*** | (0.009) | -0.784*** | (0.006) | -0.875*** | (0.001) | -0.770*** | (0.007) |
| Care-homes price | 0.541 | (0.207) | 0.634** | (0.046) | 0.542 | (0.203) | 0.791 | (0.127) | 0.873** | (0.026) | 0.787 | (0.128) |
| Pop 65+ | 1.603*** | (0.000) | 1.684*** | (0.000) | 1.583*** | (0.000) | 2.022*** | (0.000) | 2.145*** | (0.000) | 2.000*** | (0.000) |
| 2010 | -0.160** | (0.011) | -0.0659* | (0.097) | -0.131*** | (0.009) | -0.319*** | (0.001) | -0.133** | (0.043) | -0.263*** | (0.004) |
| 2011 | -0.189** | (0.049) | -0.119* | (0.057) | -0.148** | (0.046) | -0.206* | (0.087) | -0.129 | (0.120) | -0.176* | (0.069) |
| 2012 | -0.243* | (0.051) | -0.195** | (0.012) | -0.179* | (0.088) | -0.223 | (0.119) | -0.212** | (0.032) | -0.182 | (0.147) |
| 2013 | -0.467*** | (0.001) | -0.277*** | (0.002) | -0.361*** | (0.004) | -0.480*** | (0.004) | -0.289*** | (0.008) | -0.409*** | (0.008) |
| Beds spatial lag | -2.975*** | (0.002) |  |  | -2.311** | (0.015) | -4.375*** | (0.004) |  |  | -3.640** | (0.022) |
| Pop 65+ spatial lag | 5.045*** | (0.001) |  |  | 3.861** | (0.011) | 7.466*** | (0.002) |  |  | 6.199** | (0.015) |
| Constant | -45.53*** | (0.000) | -15.92*** | (0.000) | -38.48*** | (0.000) | -62.33*** | (0.000) | -18.98*** | (0.000) | -55.57*** | (0.001) |
| Error spatial lag | 0.342*** | (0.006) |  |  |  |  | 0.282* | (0.052) |  |  |  |  |
| Dep spatial lag |  |  | 0.504*** | (0.000) | 0.330*** | (0.005) |  |  | 0.489*** | (0.000) | 0.267* | (0.055) |
| R^2^ | 0.524 |  | 0.504 |  | 0.523 |  | 0.484 |  | 0.458 |  | 0.484 |  |
| Mundlak Test | 4.298 | 0.507 | 3.745 | 0.290 | 3.970 | 0.554 | 3.282 | 0.657 | 2.413 | 0.491 | 2.871 | 0.720 |
| Hausman Test | 5.619 | 0.777 | 6.858 | 0.444 | 5.359 | 0.802 | 4.496 | 0.876 | 6.121 | 0.526 | 4.216 | 0.897 |

Dependent variables are for delays officially attributed to social care. Dependent variable and continuous explanatories are in logs. *Dep spatial lag* is spatial lag of dependent variable. All models are estimated with random effects and cluster robust standard errors. SDEM: Spatial Durbin Error Model, SAR: Spatial Autoregressive Model, SDM: Spatial Durbin Model. Observations: 735 = 5x147. *p<0.1, **p<0.05, ***p<0.01

**Table A4. Delayed Discharges (all delays). Alternative Spatial Models**

|  | Patients Delayed | | | | | | Days of Delay | | | | | |
| --- | --- | --- | --- | --- | --- | --- | --- | --- | --- | --- | --- | --- |
|  | SDEM | | SAR | | SDM | | SDEM | | SAR | | SDM | |
| Care-homes beds | -0.348** | (0.016) | -0.442*** | (0.001) | -0.338** | (0.019) | -0.308* | (0.053) | -0.412*** | (0.005) | -0.297* | (0.063) |
| Care-homes price | -0.00648 | (0.981) | 0.240 | (0.278) | -0.0183 | (0.946) | -0.0323 | (0.910) | 0.222 | (0.331) | -0.0567 | (0.843) |
| Pop 65+ | 1.392*** | (0.000) | 1.468*** | (0.000) | 1.377*** | (0.000) | 1.382*** | (0.000) | 1.466*** | (0.000) | 1.365*** | (0.000) |
| 2010 | -0.0562 | (0.488) | -0.0298 | (0.273) | -0.0441 | (0.176) | -0.105 | (0.306) | -0.0440 | (0.139) | -0.0617* | (0.082) |
| 2011 | -0.0986 | (0.437) | -0.0677 | (0.145) | -0.0443 | (0.384) | -0.0524 | (0.737) | -0.0491 | (0.287) | -0.0237 | (0.636) |
| 2012 | -0.108 | (0.452) | -0.105** | (0.050) | -0.0279 | (0.682) | -0.0145 | (0.931) | -0.0689 | (0.173) | 0.0176 | (0.785) |
| 2013 | -0.196 | (0.202) | -0.143** | (0.017) | -0.0928 | (0.233) | -0.113 | (0.531) | -0.108* | (0.061) | -0.0533 | (0.481) |
| Beds spatial lag | -1.840*** | (0.003) |  |  | -1.227** | (0.050) | -2.079*** | (0.002) |  |  | -1.406** | (0.033) |
| Pop 65+ spatial lag | 2.922*** | (0.002) |  |  | 1.513 | (0.129) | 3.418*** | (0.001) |  |  | 1.758* | (0.095) |
| Constant | -26.77*** | (0.000) | -12.65*** | (0.000) | -17.89*** | (0.006) | -27.07*** | (0.000) | -11.97*** | (0.000) | -18.35*** | (0.007) |
| Error spatial lag | 0.674*** | (0.000) |  |  |  |  | 0.732*** | (0.000) |  |  |  |  |
| Dep spatial lag |  |  | 0.602*** | (0.000) | 0.590*** | (0.000) |  |  | 0.686*** | (0.000) | 0.682*** | (0.000) |
| R^2^ | 0.674 |  | 0.665 |  | 0.674 |  | 0.669 |  | 0.657 |  | 0.668 |  |
| Mundlak Test | 3.552 | 0.616 | 5.861 | 0.119 | 3.039 | 0.694 | 7.001 | 0.221 | 10.08 | 0.0179 | 6.518 | 0.259 |
| Hausman Test | 4.572 | 0.870 | 2.736 | 0.908 | 1.543 | 0.997 | 8.525 | 0.482 | 10.09 | 0.184 | 6.188 | 0.721 |

Dependent variables are for all delays. Dependent variable and continuous explanatories are in logs. *Dep spatial lag* is spatial lag of dependent variable. All models are estimated with random effects and cluster robust standard errors. SDEM: Spatial Durbin Error Model, SAR: Spatial Autoregressive Model, SDM: Spatial Durbin Model. Observations: 735 = 5x147. *p<0.1, **p<0.05, ***p<0.01

**Table A5. Patients Delayed and Days of Delay. IV Models with 2 year lag Instruments**

|  | Patients delayed | | | | Days of delay | | | |
| --- | --- | --- | --- | --- | --- | --- | --- | --- |
|  | Attributed to social care | | All delays | | Attributed to social care | | All delays | |
|  | coef | p | coef | p | coef | p | coef | p |
| Care-homes beds | -0.718*** | (0.000) | -0.452*** | (0.000) | -1.067*** | (0.000) | -0.452** | (0.012) |
| Care-homes price | 1.061*** | (0.001) | 0.271 | (0.241) | 1.684*** | (0.003) | 0.650* | (0.081) |
| Pop 65+ | 1.719*** | (0.000) | 1.482*** | (0.000) | 2.262*** | (0.000) | 1.504*** | (0.000) |
| 2011 | -0.0586 | (0.536) | -0.0437 | (0.462) | 0.0541 | (0.581) | 0.0100 | (0.843) |
| 2012 | -0.154 | (0.134) | -0.0805 | (0.238) | -0.0355 | (0.788) | -0.00390 | (0.957) |
| 2013 | -0.393*** | (0.000) | -0.161** | (0.016) | -0.311** | (0.012) | -0.0869 | (0.249) |
| Beds spatial lag | -2.390*** | (0.000) | -1.137*** | (0.007) | -3.181*** | (0.004) | -0.835 | (0.180) |
| Pop 65+ spatial lag | 4.660*** | (0.000) | 2.106*** | (0.001) | 6.584*** | (0.000) | 2.134** | (0.015) |
| Constant | -49.34*** | (0.000) | -25.25*** | (0.000) | -68.05*** | (0.000) | -27.16*** | (0.000) |
| R^2^ | 0.516 |  | 0.669 |  | 0.476 |  | 0.661 |  |
| F Test (Beds) | 674.08 | (0.000) | 556.52 | (0.000) | 202.11 | (0.000) | 128.65 | (0.000) |
| F Test (Price) | 589.55 | (0.000) | 509.45 | (0.000) | 220.33 | (0.000) | 147 | (0.000) |
| F Test (Beds spatial lag) | 6846.63 | (0.000) | 5975.83 | (0.000) | 2735.67 | (0.000) | 1858.5 | (0.000) |
| Hausman Test | 0.328 | 0.999 | 0.595 | 0.996 | 2.517 | 0.867 | 2.918 | 0.819 |

Dependent variable and continuous explanatories are in logs. All models are estimated with random effects and cluster robust standard errors.

Observations: 735 = 5x147. *p<0.1, **p<0.05, ***p<0.01. F tests are for the joint significance of the instruments in each first stage model.

The instruments are two year lag of care-homes beds, two year lag of care-homes price, and two year spatially lagged care-homes beds.

**Table A6. Patients Delayed and Days of Delay. Random effects IV Models with 1 year lag Instruments**

|  | Patients Delayed | | | | Days of Delay | | | |
| --- | --- | --- | --- | --- | --- | --- | --- | --- |
|  | Attributed to social care | | All delays | | Attributed to social care | | All delays | |
|  | coef | p | coef | p | coef | p | coef | p |
| Care-homes beds | -0.847*** | (0.007) | -0.583*** | (0.001) | -1.017** | (0.019) | -0.496*** | (0.007) |
| Care-homes price | 0.934** | (0.016) | 0.0931 | (0.698) | 1.317** | (0.013) | 0.117 | (0.647) |
| Pop 65+ | 1.941*** | (0.000) | 1.658*** | (0.000) | 2.345*** | (0.000) | 1.608*** | (0.000) |
| 2010 | -0.0986* | (0.075) | -0.0384 | (0.332) | -0.224*** | (0.004) | -0.110*** | (0.008) |
| 2011 | -0.190*** | (0.003) | -0.0936** | (0.033) | -0.208** | (0.013) | -0.0887* | (0.052) |
| 2012 | -0.326*** | (0.000) | -0.148*** | (0.004) | -0.347*** | (0.001) | -0.0899* | (0.077) |
| 2013 | -0.449*** | (0.000) | -0.172*** | (0.002) | -0.446*** | (0.000) | -0.103* | (0.066) |
| Constant | -17.86*** | (0.000) | -10.96*** | (0.000) | -20.16*** | (0.000) | -7.957*** | (0.000) |
| R^2^ | 0.489 |  | 0.662 |  | 0.442 |  | 0.655 |  |
| F Test (Beds) | 162.24 | (0.000) | 184.07 | (0.000) | 156.87 | (0.000) | 166.42 | (0.000) |
| F Test (Price) | 337.51 | (0.000) | 380.5 | (0.000) | 326.85 | (0.000) | 345.79 | (0.000) |
| Hausman Test | 2.241 | 0.945 | 2.100 | 0.954 | 1.585 | 0.979 | 4.555 | 0.714 |

Dependent variable and continuous explanatories are in logs. All models are estimated with random effects and cluster robust standard errors.

Observations: 735 = 5x147. *p<0.1, **p<0.05, ***p<0.01. F tests are for the joint significance of the instruments in each first stage model.

The instruments are one year lag of care-homes beds and one year lag of care-homes price.
